# P38 MAP Kinase Signaling Is Required for the Conversion of CD4+CD25− T Cells into iTreg

**DOI:** 10.1371/journal.pone.0003302

**Published:** 2008-10-01

**Authors:** Samuel Huber, Jörg Schrader, Gerhard Fritz, Katrin Presser, Steffen Schmitt, Ari Waisman, Stefan Lüth, Manfred Blessing, Johannes Herkel, Christoph Schramm

**Affiliations:** 1 I. Medizinische Klinik, Universitätsklinikum Hamburg-Eppendorf, Hamburg, Germany; 2 Institut für Pharmakologie und Toxikologie, Justus-Liebig-Universität, Giessen, Germany; 3 DKFZ, Ruprecht-Karls- Universität Heidelberg, Heidelberg, Germany; 4 I. Medizinische Klinik, Johannes Gutenberg-Universität, Mainz, Germany; 5 Biotechnologisches Biomedizinisches Zentrum, Universität Leipzig, Leipzig, Germany; New York University School of Medicine, United States of America

## Abstract

CD4+CD25+ regulatory T cells (Treg) are important mediators of immune tolerance. A subset of Treg can be generated in the periphery by TGF-beta dependent conversion of conventional CD4+CD25− T cells into induced Treg (iTreg). In chronic viral infection or malignancy, such induced iTreg, which limit the depletion of aberrant or infected cells, may be of pathogenic relevance. To identify potential targets for therapeutic intervention, we investigated the TGF-beta signaling in Treg. In contrast to conventional CD4+ T cells, Treg exhibited marked activation of the p38 MAP kinase pathway. Inhibition of p38 MAP kinase activity prevented the TGF-beta-dependent conversion of CD4+CD25− T cells into Foxp3+ iTreg in vitro. Of note, the suppressive capacity of nTreg was not affected by inhibiting p38 MAP kinase. Our findings indicate that signaling via p38 MAP kinase seems to be important for the peripheral generation of iTreg; p38 MAP kinase could thus be a therapeutic target to enhance immunity to chronic viral infection or cancer.

## Introduction

CD4+CD25+ Treg are crucial for the maintenance of peripheral immune tolerance and the prevention of chronic inflammation and autoimmune disease [1]. In vivo, the majority of the CD4+CD25+ Treg pool is generated in the thymus (natural or nTreg) [2]; but a significant proportion of CD4+CD25+ Treg seems to be generated in the periphery (induced or iTreg) [3], [4], [5], [6], [7], [8]. Cancer cells, as well as certain viruses are believed to take advantage of this process in that they induce CD4+CD25+ Treg in the periphery, which suppress the efficient elimination of infected or aberrant cells [9], [10], [11], [12]. Therefore, manipulation of this process could help to activate immunity to virus or cancer.

We hypothesized that interference with TGF-beta signaling could be a possible way to manipulate the peripheral generation of iTreg. TGF-beta is a pleiotropic cytokine with a prominent role in the homeostatic regulation of the immune system [13]. While the thymic generation of nTreg occurs independently from TGF-beta [14], [15], there is accumulating evidence that TGF-beta promotes the expansion of the peripheral Treg pool in vivo [5], either through direct expansion of precommitted nTreg [10], [15], [16], or through the conversion of conventional CD4+CD25− T cells into Foxp3-expressing iTreg [3], [4], [6], [8]. This process seems to depend on Smad3 activation [17]. Furthermore, TGF-beta signaling has been reported to be important for the suppressive function of nTreg [14], [15].

To identify the relevant signaling pathways involved in the TGF-beta–induced peripheral generation of iTreg, we here assessed Smad and p38 signaling in murine nTreg and iTreg. Upon binding of TGF-beta to type II and recruitment of type I receptors the major TGF-beta signal is transmitted by Smad proteins. Smad 2 and/or Smad 3 are being phosphorylated and form a complex with Smad 4, which then translocates to the nucleus in order to modulate the expression of TGF-beta regulated genes [13]. Alternatively, TGF-beta may induce activation of p38 MAP kinase through a Smad-independent signaling pathway [18]. The p38 MAP kinase belongs to the family of MAP kinases, which also includes ERK-1/2 and JNK [19]. Since mice deficient in p38 exhibit embryonic lethality [20], most analyses of p38 function in lymphocytes have relied on the use of pharmacologic inhibitors, such as SB203580 [21].

## Materials and Methods

### Animals

For all experiments age-matched FVB/N mice were used. Animal care was in accordance with the governmental and institutional guidelines. Approval was given by the local institutional committee (‘Behörde für Soziales, Familie, Gesundheit und Verbraucherschutz, Freie und Hansestadt Hamburg, Nr. 97/06’).

### Lymphocyte separation, cell culture and cell proliferation assays

The isolation of CD4+CD25− and CD4+CD25+ T cells from the spleens of 7 to 8 weeks old FVB/N mice by MACS and FACS was performed essentially as described [15], [22]. For the conversion of CD4+CD25− T cells into iTreg, CD4+CD25− T cells were activated with 2 µg/ml plate bound CD3 mAb and 2 µg/ml soluble CD28 mAb (BD, Heidelberg, Germany) in the presence of 2 ng/ml hTGF-beta1 (R&D Systems, Mineapolis, MN). The expression of Foxp3 and CD25 was assessed by flow cytometry as a marker of conversion. To test the suppressive capacity of nTreg or iTreg, freshly isolated CD4+CD25− T cells from spleen of wild type animals as responder cells were labelled with CFSE (2 µM, 5 min.; 5-,6- carboxyfluorescein diacetate, succinimidyl ester; Molecular Probes, Leiden, The Netherlands), and stimulated with allogeneic, irradiated APC and soluble CD3 mAb (3 µg/ml) for 4 days in the presence or absence of nTreg or the whole cell suspension gained by in vitro conversion assays at ratios indicated (responder+suppressor×10^5^). CFSE dilution as a marker for proliferation was measured using flow cytometry. SB203580, SB202190, or SP600125 (all 10 µM) or PD98059 (50 µM) in 10% DMSO/PBS was added to the culture medium every 12 h (all from Calbiochem, Darmstadt, Germany). DMSO was added to control cultures at equivalent concentrations.

### Flow cytometry

For flow cytometric analyses, cells were stained with Foxp3-PE/APC (eBioscience, San Diego, CA) and CD25 FITC (BD) according to the manufacturers instructions. Flow cytometry was performed with a FACSCalibur using CellQuest software or with a FACSCanto (BD Biosciences). At least 1×10^4^ cells were analyzed.

### Western blot analysis

Cell lysates were prepared by homogenization of snap frozen cell pellets in PBS containing 1% TritonX-100 supplemented with NaF (10 mM), EDTA (2 mM), benzamidine (10 mM), PMSF (1 mM), leupeptin (1 µg/ml), Na_3_VO_4_ (2 mM) and aprotinin (1,5 µg/ml). Protein content was measured by Bradford-Assay and 10 µg total protein was loaded onto 12% SDS/Page. Proteins were blotted onto nitrocellulose membranes (Schleicher&Schuell, Dassel, Germany), blocked in 5% dry non-fat milk and probed with primary antibodies and appropriate HRP-conjugated secondary antibodies. Detection was performed using an ECL kit (Roth, Karlsruhe, Germany).

All chemicals were purchased from Sigma (St. Louis, IL, USA), if not otherwise stated. All antibodies were purchased from Cell Signaling (Danvers, MA, USA), except for p-Erk and Actin (Santa Cruz, CA, USA), Smad7 (R&D Systems, Minneapolis, MN, USA) and FoxP3 (eBioscience, San Diego, CA). Secondary HRP-conjugated anti-mouse and anti-rabbit antibodies were supplied by Cell Signaling (Danvers, MA, USA).

### Statistical analysis

Values are presented as mean +/− SEM per group. The non-parametric Mann-Whitney U test was used and a p<0.05 considered as significant. SPSS statistical software (SPSS inc., Chicago, USA) was used for analysis.

## Results

### Increased activity of the p38 MAP kinase pathway in freshly isolated CD4+CD25+ T cells

To analyze which of the possible TGF-beta-induced signaling pathways may be of relevance for Treg in vivo, we studied the phosphorylation of Smad 2/3 and p38, ERK or JNK MAP kinases in freshly isolated murine CD4+CD25+ and CD4+CD25− T cells.

**Figure 1:**
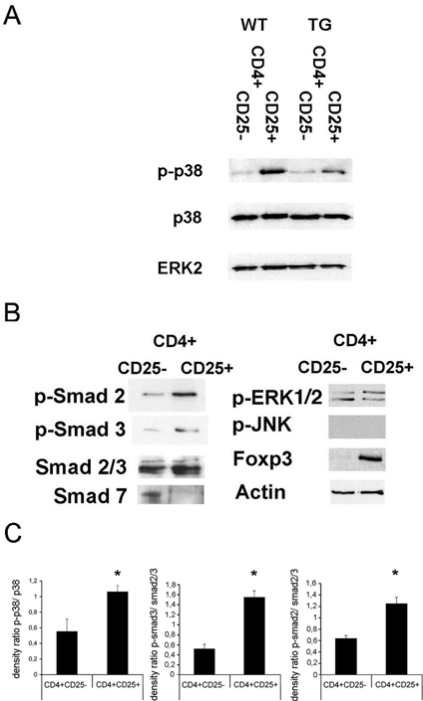
Western blot analysis of MAP kinase and Smad phosphorylation in CD4+CD25− and CD4+CD25+ T cells (2–4×10^6^ cells). A: Analysis of p38 and ERK2 expression as well as p38 phosphorylation in CD4+CD25− and CD4+CD25+ T cells freshly isolated from spleen of wild type mice or transgenic mice overexpressing a dominant negative TGF-beta type II receptor in T cells. B: Analysis of Smad 2/3, Smad 7, Foxp3, actin, p-Smad 2, p-Smad 3, p-JNK and p-ERK1/2 from CD4+CD25− and CD4+CD25+ T cells freshly isolated from spleen of wild type animals. C: Densitometry and ratio of phosphorylated to unphosphorylated p38 and Smad 2/3 (p<0.05). The experiments were at least repeated three times giving similar results.

The amounts of p38 MAP kinase appeared to be similar both in CD4+CD25+ and CD4+CD25− T cells (Figure 1A; WT). However, the phosphorylation of p38 MAP kinase in CD4+CD25+ T cells was strongly increased as compared to CD4+CD25− T cells (Figure 1A; WT). Of note, the phosphorylation of p38 MAP kinase was decreased in transgenic CD4+CD25+ T cells that overexpress a dominant negative TGFbeta type II receptor, indicating at least partial TGF-beta-dependence of the p38 signal (Figure 1A; TG). We did not find any significant differences in the phosphorylation of JNK or ERK between freshly isolated CD4+CD25− and CD4+CD25+ T cells (Figure 1B). As compared to CD4+CD25− T cells, the phosphorylation of Smad 2 and Smad 3 was increased whereas the inhibitory Smad 7 was found to be strongly downregulated in CD4+CD25+ T cells (Figure 1 B+C). In order to adjust for potential differences in the amount of Smad 2- or 3-expression, we determined the ratio of phosphorylated to unphosphorylated protein in four independent experiments, and found that activation of Smad 2 and 3 and of p38 was significantly increased in the CD4+CD25+ T cells, as compared to the CD4+CD25− T cells (Figure 1C).

### TGF-beta1 activates p38 MAP kinase in naïve T cells and inhibition of this signaling blocks the conversion of CD4+CD25− T cells into Foxp3-expressing, TGF-beta-induced iTreg in vitro

**Figure 2:**
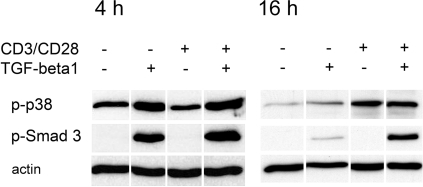
TGF-beta1 activates Smad 3 and p38 MAP kinase in CD4+CD25− T cells. Freshly isolated CD4+CD25− T cells (4×10^6^) were cultured with or without TGF-beta1 for 4 or 16 hours. Cells were activated with plate bound anti-CD3 mAb (2 µg/ml) and soluble anti-CD28 mAb (2 µg/ml) as indicated. Smad 3 and p38 activation were measured using Western blot. The experiments were repeated three times giving similar results.

It has been shown that TGF-beta1 induces Foxp3 transcription in peripheral naïve CD4 T cells [3]. This conversion to iTreg seems to depend on Smad 3 activation [17]. We therefore first analyzed TGF-beta1 signaling pathways in freshly isolated CD4+CD25− T cells. In the absence of activating signals and TGF-beta1, spontaneous activation of p38 MAP kinase as well as Smad 3 was observed (Figure S 1), which may have been caused by the isolation procedure and/or cytokines carried over into the culture. The spontaneous Smad 3 activation lasted for at least 2 hours, that of p38 lasted for at least 4 hours (Figure S 1). Addition of TGF-beta1 in the absence of activating signals induced the activation of both Smad 3 and p38, detectable at 4 hours and 16 hours of culture (Figure 2). At 16 hours, but not at 4 hours of culture, T cell activation by CD3 and CD28 antibody enhanced the TGF-beta1-induced p38 and Smad 3 activation (Figure 2). T cell activation in the absence of TGF-beta1 did not induce Smad 3 activation, but p38 phosphorylation, weakly at 4 hours and strongly at 16 hours of culture (Figure 2). Thus, in addition to Smad signaling, TGF-beta1 seemed to induce early p38 activation, detectable at 4 hours of culture (Figure 2).

**Figure 3:**
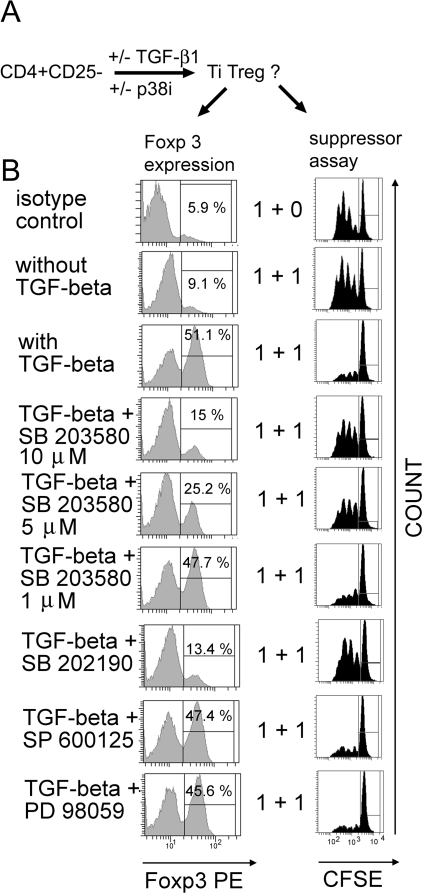
Signaling via p38 MAP kinase is required for the in vitro conversion of CD4+CD25− T cells into TGF-beta1-induced Foxp3+ Treg (Ti Treg). CD4+CD25− T cells were activated with plate bound anti-CD3 mAb (2 µg/ml) and soluble anti-CD28 mAb (2 µg/ml) for 4 days in the presence or absence of TGF-beta1 (2 ng/ml). Kinase inhibitors were added every 12 h (SB203580 (10 µM), SP600125 (10 µM), PD89059 (50 µM)). A: Schematic experimental procedure. Ti Treg: TGF-beta-induced Treg. B: Foxp3-expression and in vitro suppressor assay using CD4+CD25− CFSE-labelled responder T cells isolated from spleen and suppressor T cells generated by in vitro conversion as described above. Cells were washed three times before adding to the culture. Responder and suppressor cells were added at the indicated ratios [×10^5^]. Data are representative of three independent experiments using SP600125, PD89059, SB202190 and five independent experiments using SB203580.

We therefore next analyzed the role of p38 MAP kinase signaling for the conversion of CD4+CD25− T cells into Foxp3-expressing iTreg in vitro (Figure 3A). CD4+CD25− T cells were cultured for four days in the presence or absence of TGF-beta1. The role of p38 signaling was investigated by adding the specific p38 inhibitors SB203580 or SB202190 to the cultures twice daily. Alternatively, ERK inhibitor PD98059 or JNK inhibitor SP600125 was added to the culture. The rates of conversion into iTreg were determined by flow cytometric analysis of Foxp3-expression and assessment of the functional suppressive activity of the induced cells in vitro (Figure 3B).

**Figure 4:**
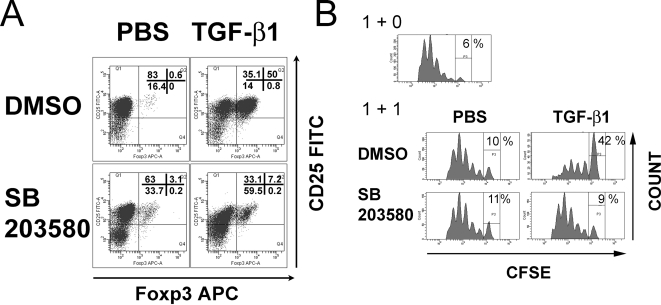
Converted cells express CD25 and Foxp3 and are functional in vitro. A: Representative CD25-/Foxp3-expression after in vitro conversion into iTreg in the presence or absence of SB203580. B: In vitro suppressor assay using CD4+CD25− CFSE-labelled responder T cells isolated from spleen and suppressor T cells generated by in vitro conversion as described. Responder and suppressor cells were added at a ratio of 1∶1. Suppressor cells were washed three times before addition to the assay.

In the absence of inhibitor, CD4+CD25− T cells cultured with TGF-beta1 acquired suppressive function and showed an increased Foxp3-expression (Figure 3B). In contrast, the presence of either p38 inhibitor dose-dependently blocked the TGF-beta-induced conversion into iTreg, as seen by a reduced suppressive activity and Foxp3-expression (Figure 3B). A representative analysis of CD25− and Foxp3-expression after the TGF-beta induced in vitro conversion in the presence or absence of the inhibitor SB203580 is shown in Figure 4. The inhibitor did not compromise viability of the cells, as determined by staining with propidium iodide (91.5% negative with inhibitor vs. 90.5% negative without inhibitor), and similar cell numbers could be obtained after culture (on average 6.8×10^5^ with TGF-beta1 and inhibitor vs. 7.1×10^5^ with TGF-beta1 and without the inhibitor).

At higher concentrations than used in our experiments, the p38 inhibitors have been reported to inhibit other MAP kinases [21], [23]. To exclude that the blockade of Foxp3-induction by the p38 inhibitor was not mediated by p38, but other MAP kinases, we also tested inhibitors of ERK and JNK in the same assay. However, these inhibitors had no effect on the conversion of CD4+CD25− T cells into Foxp3+ iTreg (Figure 3B).

**Figure 5:**
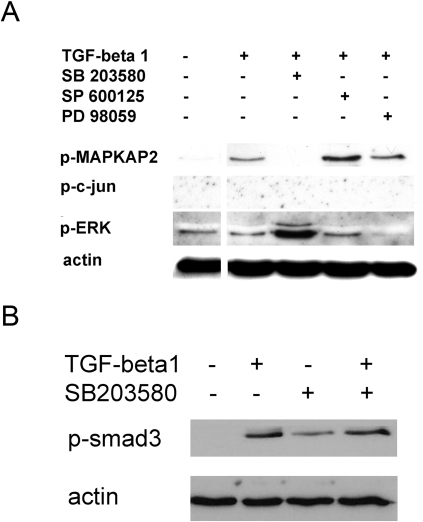
p38 MAP kinase inhibitor SB203580 specifically inhibits the phosphorylation of p38 MAP kinase downstream target MAPKAP2. A: CD4+CD25− T cells were cultured with TGF-beta1 (2 ng/ml) and SB203580 (10 µM), SP600125 (10 µM), or PD89059 (50 µM) for 45 min. Control cells were cultured without TGF-beta1. B: CD4+CD25− T cells were cultured for 4 hours with TGF-beta1 (2 ng/ml) and SB203580 (10 µM). P-MAPKAP2, p-ERK, p-c-jun, p-Smad 3 and actin were determined using Western blot. Results are representative of two independent experiments.

To further confirm the specificity of inhibition by the inhibitor SB203580 and the control inhibitors of JNK (SP600125) and ERK (PD98059), we analysed the activities of the p38, JNK and ERK pathways during in vitro conversion into iTreg (Figure 5A). We found that the p38 inhibitor SB203580 selectively blocked the phosphorylation of MAPKAP2, which is a downstream target of p38 MAP kinase (Figure 5A). Accordingly, PD98059 blocked the phosphorylation of ERK, but not MAPKAP2. The JNK pathway seemed to be only minimally activated in vitro (data not shown) and we could therefore not detect any phosphorylation of the JNK downstream target c-jun, making it difficult to prove a selective effect of SP600125 on the JNK pathway (Figure 5A). Of note, the p38 inhibitor SB203580 did not block Smad signaling, but rather seemed to induce Smad 3 activation itself (Figure 5B).

### p38 MAP kinase signaling is not required for the suppressive function of nTreg in vitro

**Figure 6:**
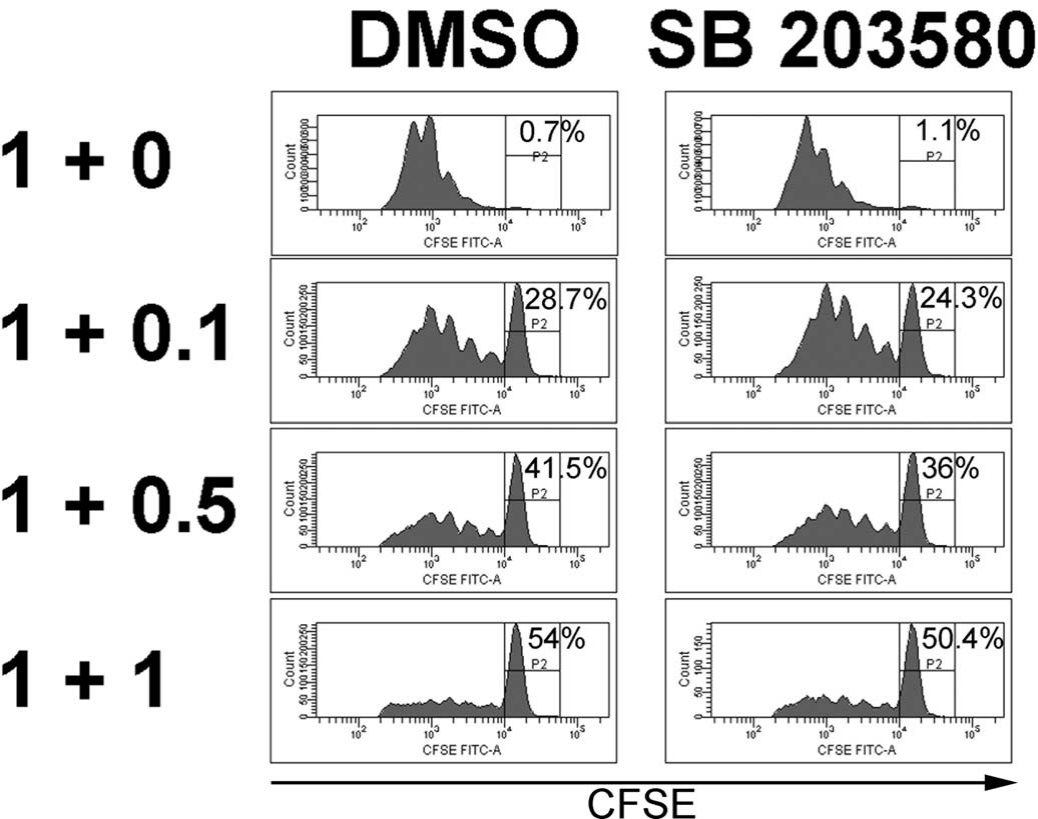
P38 MAP kinase signaling is not required for the in vitro suppressive function of CD4+CD25+ T cells. Freshly isolated CD4+CD25− T cells from spleen were labelled with CFSE and cultured for four days under CD3 stimulation (3 µg/ml) in the presence of allogeneic CD3− spleen cells with or without CD4+CD25+ T cells freshly isolated from wild type mice, added at the indicated cell numbers (responder+suppressor cells×10^5^). DMSO or SB203580 (10 µM) were added to the culture twice daily as indicated. CFSE dilution was measured by flow cytometry after 4 days of coculture. Data are representative of four independent experiments.

The above findings indicate that p38 MAP kinase could be a therapeutic target for preventing the peripheral generation of iTreg in the context of malignancies or chronic viral infection. However, a complete inhibition of Treg suppressive function could have adverse effects and induce autoimmune disease. We therefore tested whether p38 inhibition may inhibit the suppressive function of nTreg. To that end, the p38 inhibitor SB203580 was added to an in vitro suppressor assay twice daily (Figure 6). We did not find a significant difference in the suppressive capacity of nTreg with or without p38 inhibitor (1+1, % of undivided cells: 54% (DMSO) vs. 50% (SB203580)). Similar results were obtained using another p38 MAP kinase inhibitor (SB202190, not shown). These findings indicate that the in vitro suppressive function of established nTreg is not blocked by inhibition of the p38 MAP kinase pathway.

## Discussion

The conversion of CD4+CD25− T cells into iTreg may be an important mechanism in the dynamic regulation of immune responses in vivo and the elucidation of its mechanisms may open new targets for therapeutic intervention. We were therefore interested in signaling pathways involved in peripheral Treg generation. It has been previously shown that TGF-beta1 induces Foxp3-expression in peripheral naïve CD4 T cells in vitro and in vivo [3], [4], [22]. This conversion to iTreg seems to depend on the main TGF-beta signaling pathway via activation of Smad 3 and concomitant downregulation of inhibitory Smad 7 as well as suboptimal TCR stimulation [17], [24]. In addition to the Smad cascade we have here analyzed p38 activation as an alternative TGF-beta induced signaling pathway in Treg. We found an increased activation of p38 MAP kinase in freshly isolated nTreg as compared to CD4+CD25− T cells. This effect was at least partially mediated via TGF-beta, as demonstrated in transgenic CD4+CD25+ T cells with impaired TGF-beta signaling. In addition to p38, the phosphorylation of Smad 2 and 3 was upregulated and the expression of Smad 7 reduced in nTreg as compared to CD4+CD25− T cells, giving support to the notion that active TGF-beta signaling is required for Treg homeostasis in vivo. These findings suggested that the p38 signaling pathway, in addition to the Smad pathway, seemed to be spontaneously activated in nTreg.

We then investigated, whether the p38 MAP kinase pathway may be involved in the conversion of naive T cells into iTreg. P38 and Smad 3 were found to be spontaneously activated in freshly isolated CD4+CD25− T cells, probably induced by the cell isolation procedure. We therefore analyzed the cells after a resting period of 4 hours. This may in part explain the differences observed in comparison to previously published reports on the effect of costimulation on p38 activation in T cell lines [25], [26]. After cells had rested for four hours, TGF-beta was demonstrated to induce phosphorylation of p38, whereas costimulation with anti-CD3/CD28 had no effect on the activation of p38 at this time point. At 16 hours of culture, costimulation alone was able to strongly induce p38 activation, as has been reported for primary mouse T cells [27]. The differences in timing and strength of activation may relate to the fact that different time points were analyzed and that complete medium supplemented with FCS, which contains growth factors such as TGF-beta, were used in the study by Zhang et al. [27].

We here report that TGF-beta1-induced conversion of CD25− T cells into Foxp3-expressing iTreg could be suppressed by the addition of pharmacological inhibitors of p38. These findings are in accordance with a recently published study reporting impaired DC-induced conversion into iTreg after inhibition of p38 with SB203580 [28] and suggest that p38 signaling is of functional relevance for the generation of iTreg.

It has been reported that using high concentrations of SB203580 kinases other than p38 may be inhibited as well [21], [23]. At the rather low concentrations used in our experiments we could demonstrate specific inhibition of p38 downstream target MAPKAP2 using SB203580. Interestingly, the phosphorylation of Erk seemed to be increased by inhibiting p38, as has been reported before [29]. The three MAP cascades may interact and the inhibition of one may therefore enhance the activation of the other as suggested for p38 and JNK [30]. In a similar manner, p38 and Smad 3 signaling pathways may interact as it appeared that inhibition of p38 augmented phosphorylation of Smad 3. This has to our knowledge not been reported before and clearly requires further investigation.

From the data presented (Figure 4) it may appear that SB203580 affects CD25-expression. We therefore analyzed cell activation markers and proliferation of T cells cultured in the presence of the inhibitor. No differences in CD44-expression (Figure S 2) or in cell numbers between cultures with or without the inhibitor could be detected. Also, the proliferation of CD25− T cells was not affected by SB203580 (Figure 6), arguing against an effect on overall T cell activation.

Pharmacological inhibition of p38 MAP kinase did not affected the in vitro suppressive function of established nTreg. If confirmed in vivo, it seems unlikely that the clinical use of p38 inhibitors may lead to the induction of autoimmune disease. Indeed, such inhibitors are being tested in clinical trials for the treatment of autoimmune diseases, in which they appear to be safe [31]. However, our and the recently published [28] findings that such p38 inhibitors may have the potential to interfere with the peripheral generation of iTreg indicate that the treatment of autoimmune diseases may not be the appropriate indication for these inhibitors. It remains to be seen whether inhibition of the p38 MAP kinase pathway in vivo may serve the therapy of chronic infection or cancer.

In conclusion our findings indicate that signaling via p38 MAP kinase, in addition to Smad 3 signaling is required for the in vitro conversion of CD4+CD25− T cells into iTreg and thus may be important for the regulation of the peripheral Treg pool. p38 MAP kinase may be a potential therapeutic target for preventing the induction of iTreg by malignant or infected cells in vivo. Although there was no effect of p38 inhibition on the in vitro suppressive capacity of nTreg, the effects and potential risks of p38 inhibition in vivo require further study.

## Supporting Information

Figure S1 Time course of p38 and Smad 3 activation in CD4+CD25− T cells analyzed ex vivo . Freshly isolated cells (4×10^6^) were cultured with or without TGF-beta1 (2 ng/ml) for 15 to 240 minutes. Cells were activated with plate bound anti-CD3 mAb (2 µg/ml) and soluble anti-CD28 mAb (2 µg/ml) as indicated. Smad 3 and p38 activation were measured using Western blot.
(6.30 MB TIF)

Figure S2 Representative CD44− and Foxp3-expression after the in vitro conversion of CD4+CD25− T cells into iTreg in the presence or absence of TGF-beta1 (2 ng/ml) and SB203580 (10 µM). Mean fluorescence intensity of CD44-FITC is given.
(9.98 MB TIF)
