# Supplementary figures and images for: P38 MAP Kinase Signaling Is Required for the Conversion of CD4+CD25− T Cells into iTreg
Source: PLoS One. 2008 Oct 1;3(10):e3302. doi: 10.1371/journal.pone.0003302 (PMC2553190; doi:10.1371/journal.pone.0003302)

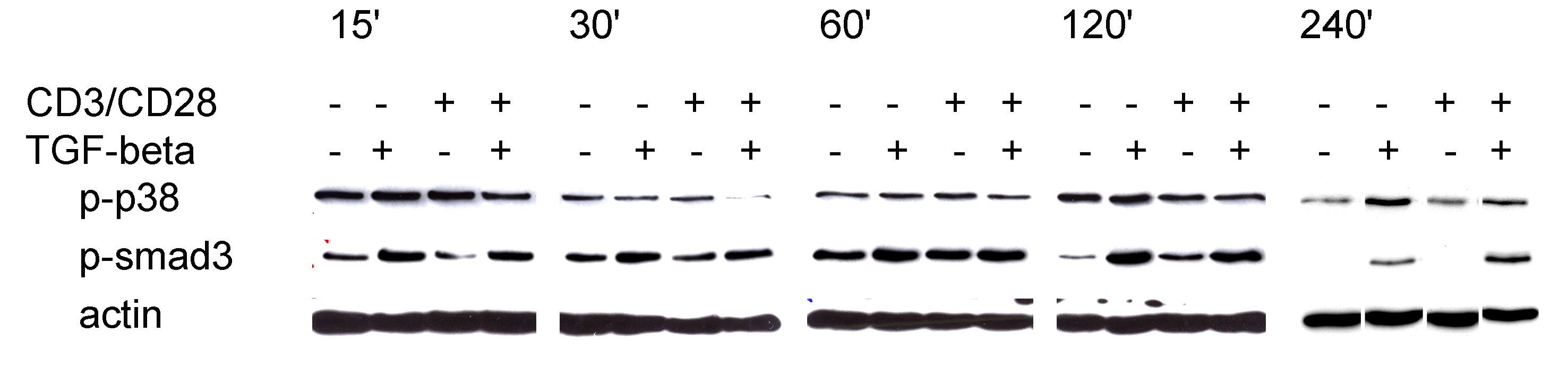

Supplement: Figure S1 — Time course of p38 and Smad 3 activation in CD4+CD25− T cells analyzed ex vivo . Freshly isolated cells (4×10^6^) were cultured with or without TGF-beta1 (2 ng/ml) for 15 to 240 minutes. Cells were activated with plate bound anti-CD3 mAb (2 µg/ml) and soluble anti-CD28 mAb (2 µg/ml) as indicated. Smad 3 and p38 activation were measured using Western blot. (6.30 MB TIF) [file pone.0003302.s001.tif]

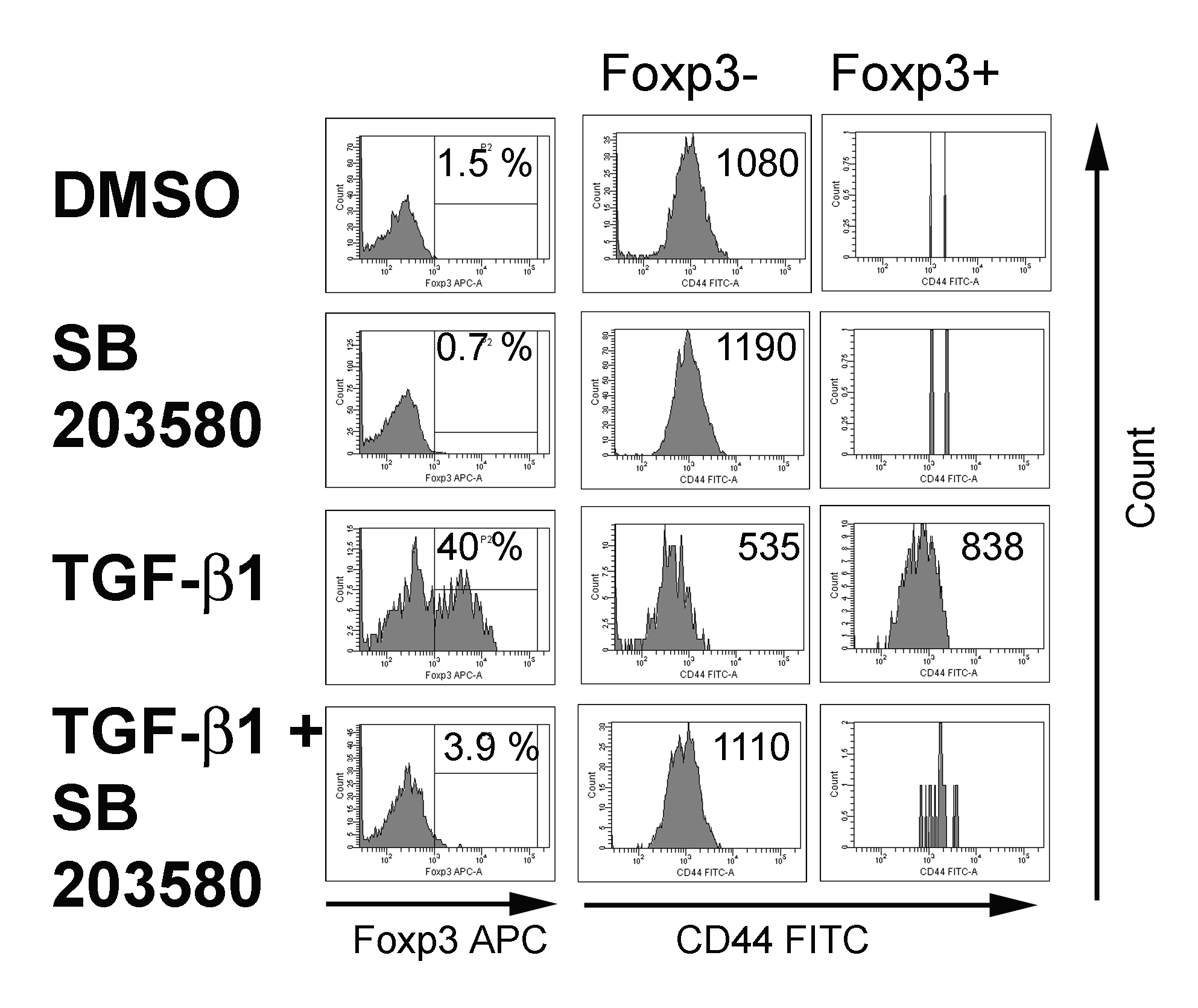

Supplement: Figure S2 — Representative CD44− and Foxp3-expression after the in vitro conversion of CD4+CD25− T cells into iTreg in the presence or absence of TGF-beta1 (2 ng/ml) and SB203580 (10 µM). Mean fluorescence intensity of CD44-FITC is given. (9.98 MB TIF) [file pone.0003302.s002.tif]
